# Extraction Optimization for Obtaining *Artemisia capillaris* Extract with High Anti-Inflammatory Activity in RAW 264.7 Macrophage Cells

**DOI:** 10.1155/2015/872718

**Published:** 2015-05-14

**Authors:** Mi Jang, Seung-Weon Jeong, Bum-Keun Kim, Jong-Chan Kim

**Affiliations:** ^1^Korea Food Research Institute, 1201-62 Anyangpangyo-ro, Bundang-gu, Seongnam-si, Gyeonggi-do 463-746, Republic of Korea; ^2^Department of Oriental Medicinal Material and Processing, College of Life Science, Kyung Hee University, 1732 Deogyeong-daero, Giheung-gu, Yongin-si, Gyeonggi-do 446-701, Republic of Korea

## Abstract

Plant extracts have been used as herbal medicines to treat a wide variety of human diseases. We used response surface methodology (RSM) to optimize the *Artemisia capillaris* Thunb. extraction parameters (extraction temperature, extraction time, and ethanol concentration) for obtaining an extract with high anti-inflammatory activity at the cellular level. The optimum ranges for the extraction parameters were predicted by superimposing 4-dimensional response surface plots of the lipopolysaccharide- (LPS-) induced PGE_2_ and NO production and by cytotoxicity of *A. capillaris* Thunb. extracts. The ranges of extraction conditions used for determining the optimal conditions were extraction temperatures of 57–65°C, ethanol concentrations of 45–57%, and extraction times of 5.5–6.8 h. On the basis of the results, a model with a central composite design was considered to be accurate and reliable for predicting the anti-inflammation activity of extracts at the cellular level. These approaches can provide a logical starting point for developing novel anti-inflammatory substances from natural products and will be helpful for the full utilization of *A. capillaris* Thunb. The crude extract obtained can be used in some *A. capillaris* Thunb.-related health care products.

## 1. Introduction

Plant extracts have been used as herbal medicines to treat a wide variety of human diseases. The herbal products today symbolize safety in contrast to synthetics, which are regarded as unsafe to humans and the environment [1]. The use of herbal and natural products in East Asian countries is increasing because of their pharmacological or biological activities [2]. Among the numerous herbs used in Oriental medicine,* Artemisia capillaris* Thunb. is one of the earliest and most important edible crude herbs used for medicinal purposes in Korea, China, and Japan.* A. capillaris* has been widely used as a hepatoprotective, analgesic, and antipyretic agent [3]. Many researchers have studied its various biological activities, such as anti-inflammatory [4], antioxidant [5], anticarcinogenic [6], and antimicrobial [3] properties.

Inflammation is a multistep process mediated by activated inflammatory and immune cells, including macrophages and monocytes [7], and comprises a complex series of reactions regulated by a cascade of cytokines, growth factors, nitric oxide (NO), and prostaglandins (PGs) produced by active macrophages [8]. Inflammation is one of the most important defense mechanisms, but prolonged inflammation contributes to the pathogenesis of many inflammatory diseases, including bronchitis [9], gastritis [10], inflammatory bowel disease [11], multiple sclerosis [12], and rheumatoid arthritis [13]. The employment of a variety of anti-inflammatory agents may help in the therapeutic treatment of pathologies associated with inflammation. The development and utilization of more effective anti-inflammatory agents of natural origin are therefore required.

Extraction is the first critical stage in the preparation of plant formulations. The crude extracts directly obtained from plants can be used as a remedial agent or the crude part can be further fractionated and purified by chemicals and solvents. Overall, the crude extracts finally lead to herbal drugs, which all have traditional medicinal value. Therefore, the standardization of extracts and extraction methods are important in the field of phytochemistry [14]. Modern methods of extraction are effective in advancing the development of traditional herbal remedies [15]. Response surface methodology (RSM) has been widely used to optimize extraction conditions such as temperature, extraction time, and concentration of solvents. RSM consists of mathematical and statistical techniques used to develop an adequate functional relationship between a response of interest and some independent variable [16].

With the increasing demand for herbal medicinal products and natural products for health care all over the world, herbal manufacturers aim for the most appropriate extraction technologies to produce extracts of defined quality with the lowest batch-to-batch variation, which can also help in the scaling-up of extraction. To have a complete understanding of the bioactivity of crude extracts, it is necessary to optimize the extraction methodology to achieve the broadest possible range of phytochemicals [17]. The objective of the present study was to apply the RSM approach to optimize the extraction temperature, extraction time, and ethanol concentration to maximize the anti-inflammatory activities from* A. capillaris* Thunb. at the cellular level. The crude extract obtained can be used in some* A. capillaris* Thunb.-related health care products. Thus, the results obtained will be helpful for the full utilization of* A. capillaris* Thunb.

## 2. Materials and Methods

### 2.1. Plant Materials and Extract Preparation

In March 2013, whole plants of* A. capillaris* Thunb. were obtained from the Department of Oriental Pharmacy, Kyung Hee Medical Center, Seoul, Korea. Voucher specimens of the plant materials are kept in our laboratory (Korea Food Research Institute, Gyeonggi, Korea) for further reference. The dried sample was ground in a blender to obtain a fine powder (particle diameter size: 500–850 *μ*m). Five grams of dried* A. capillaris* Thunb. powder was extracted by 100 mL of different ethanol concentrations at the required temperature and duration. Each extract was filtered using filter paper (Whatman number 4). The ethanol was removed under reduced pressure by rotary evaporation, and the water residue was removed by lyophilization. For testing, the extracts were dissolved in phosphate-buffered saline and diluted to the desired concentrations.

### 2.2. Cell Culture

RAW 264.7 macrophages were obtained from the Korean Cell Line Bank (KCLB, Seoul, Korea) and were maintained in RPMI 1640 medium (Gibco-BRL, Grand Island, NY, USA) containing antibiotics (100 units/mL penicillin A and 100 *μ*g/mL streptomycin) and 10% heat-inactivated fetal bovine serum (Gibco-BRL, USA) at 37°C in a 5% CO_2_ incubator.

### 2.3. MTT Assay

The viability of the cells was determined colorimetrically by using the MTT assay as described by Denizot and Lang [18] with some modification. The RAW 264.7 cells were seeded in a 96-well plate at a density of 5 × 10^5^ cell mL^−1^ and were treated with various concentrations of the extracts for 24 h. MTT solution (20 *μ*L of 5 mg/mL MTT in phosphate-buffered saline) was added to each well, and the cells were incubated for 2 h. After the supernatants were aspirated, the formazan crystals in each well were dissolved in 100 *μ*L of dimethyl sulfoxide (DMSO) and the optical density (OD) of cells at 570 nm was measured using a microplate reader (Bio-Rad, Hercules, CA, USA). The OD of the samples was compared to that of the LPS-untreated control to obtain the percentage viability.

### 2.4. Anti-Inflammatory Activity

The anti-inflammatory activities of the extracts were measured using the NO and PGE_2_ assays. Both assays have been widely used to determine the anti-inflammatory activity of medicinal plants. These methods were selected because they are based on different principles and because they are widely used. NO is a well-known proinflammatory mediator that is involved in various physiological and pathological processes. Recently, the suppression of NO production has been emphasized as a new pharmacological strategy for the treatment of inflammation-related diseases [19]. PGE_2_ is a key inflammatory mediator and stimulates cytokine generation and vasodilation and mediates fever and pain [20]. These two mediators are believed to be adequate to assess the anti-inflammatory activity of* A. capillaris* Thunb. extract.

### 2.5. Measurement of NO Production

The NO level in the cultured medium was determined by the Griess reaction [21]. The cells were pretreated with the indicated concentrations of the extracts for 2 h and then were induced with a 1 *μ*g/mL concentration of LPS for an additional 22 h. Supernatant from each well (100 *μ*L) was mixed with 100 *μ*L of Griess reagent in a separate 96-well plate. After incubation for 15 min at room temperature, the OD was measured at 540 nm using a microplate reader. NO production by the extract-treated cells is presented as a percentage of the NO production in the LPS-induced control.

### 2.6. Measurement of PGE_2_ Production

RAW 264.7 cells were incubated with LPS (1 *μ*g mL^−1^) in the presence or absence of the samples for 24 h. The samples were analyzed using PGE_2_ enzyme immune assay (EIA) kit (R&D Systems, Minneapolis, MN), according to the manufacturer's protocol. PGE_2_ concentrations in the supernatants were determined by comparison with a standard curve. PGE_2_ production by the extract-treated cells is presented as the percentage of PGE_2_ production in the LPS-induced control.

### 2.7. Experimental Design

The RSM was used to optimize extraction conditions and monitor the extraction characteristics. The experimental design was a central composite design (CCD). The 3 independent variables were extraction temperature (*X*_1_), ethanol concentration (*X*_2_), and extraction time (*X*_3_), and the response variables were LPS-induced PGE_2_ (*Y*_1_) and NO (*Y*_2_) production and cytotoxicity (*Y*_3_) in RAW 264.7 cells. Each independent variable to be optimized was coded at 5 levels (−1.682, −1, 0, 1, and 1.682) with 20 runs, including 6 replicates at the central point (Table 1). Experimental data were analyzed using the Statistical Analysis System (SAS) program (SAS Inst. Inc., Cary, NC, USA) and fitted to a second-order polynomial regression model containing the coefficient of linear, quadratic, and two-factor interaction effects. The model equation for the response of the 3 independent variables was

(1) Yn=β0+β1X1+β2X2+β3X3+β11X12+β22X22+β33X32+β1β2X1X2+β1β3X1X3+β2β3X2X3.

In this model, *Y*_*n*_ is the predicted response variable; *β*_0_ is the constant coefficient; *β*_1_, *β*_2_, and *β*_3_ are the regression coefficients for the linear effect terms; *β*_11_, *β*_22_, and *β*_33_ are the quadratic effect terms; and *β*_1_ *β*_2_, *β*_1_ *β*_3_, and *β*_2_ *β*_3_ are the interaction effect terms. The adequacy of the model was predicted through regression analysis (*R* ^2^ and adjusted *R* ^2^) and analysis of variance (ANOVA) (*P* < 0.05). The relationship between the independent variables (*X*_1_, *X*_2_, and *X*_3_) and the response variables (*Y*_1_, *Y*_2_, and *Y*_3_) was demonstrated through four-dimensional response surface plots generated using the Wolfram Mathematica software (Wolfram Research, Inc., Champaign, Illinois, USA) [22].

### 2.8. Prediction and Verification of Optimum Extraction Conditions

The optimum ranges of the extraction conditions were predicted by superimposing the response surfaces of the LPS-induced PGE_2_ and NO production, which are the representative inflammatory factors, and by cytotoxicity* in vitro*. The optimum extraction conditions were verified by comparing the experimental values with the predicted values.

### 2.9. HPLC Analysis

The analytical HPLC system employed consisted of a JASCO high-performance liquid chromatograph coupled with a UV-Vis multiwavelength detector (MD-910 JASCO). HPLC analysis operated under the following conditions: YMC-Pack ODS-AM column (250 mm × 4.6 mm i.d. and particles of 5 *μ*m) (YMC, Japan), column oven temperature 35°C, and detection 285 nm. The gradient solvent system consisted of 0.1% acetic acid in water (solvent A) and 0.1% acetic acid in acetonitrile/water (solvent B) as follows: 0-1 min, 12% B; 1–18 min, 22% B; 18–28 min, 28% B; 28–35 min, 38% B; 35–48 min, 48% B; 48–54 min, 68% B; 54–60 min, 100% B; 60–67 min, 12%. The flow rate was 1.0 mL min^−1^  and the injection volume was 10 *μ*L. The identification of each compound was based on a combination of retention time and spectral matching.

## 3. Results and Discussion

The effects of three independent process variables—extraction temperature (*X*_1_, 30–90°C), ethanol concentration (*X*_2_, 0–100%), and extraction time (*X*_3_, 1–11 h)—were investigated and CCD was applied to determine the optimal combination of the factors. The three responses of interest were LPS-induced PGE_2_, NO production, and cytotoxicity. The results of 20 runs by using the CCD design are shown in Table 1, which include the coded matrices for design conditions and corresponding results of RSM experiments.

### 3.1. Cell Viability

We examined the cytotoxic effects of* A. capillaris* Thunb. extracts on RAW 264.7 cells and found that none of the extracts affected cell viability at 5, 10, or 30 *μ*g/mL after 24 h. However, the extracts inhibited cell viability at 50 *μ*g/mL (data not shown). Thus, a concentration of 30 *μ*g/mL was used to treat the cells in the following experiments.

### 3.2. Optimization of LPS-Induced PGE_2_ Production

PGE_2_ is a mediator of active inflammation and a bioactive lipid that can elicit a wide range of biological effects associated with inflammation and cancer. It plays a critical role in guiding and governing various aspects of the inflammatory response. The role of PGE_2_ in driving acute inflammation is well established [23].

### 3.3. Model Fitting and Statistical Analysis

Table 2 shows the results of fitting quadratic models to the data. The results of ANOVA indicate that the contribution of the quadratic model was significant. A quadratic regression model for LPS-induced PGE_2_ production was obtained from the experimental data, as shown by using

(2) PGE2=173.194583−2.523157X1−0.289006X2−11.023825X3+0.019932X12+0.008825X22+0.660889X32−0.007467X1X2+0.072940X1X3−0.029131X2X3.

An ANOVA was performed to check the adequacy of the suggested models and identify the significant factors; a statistical summary is given in Table 2. The model *F*-value of 11.56 for LPS-induced PGE_2_ production implies that the model is statistically significant. There was only a 0.03% chance that a model *F*-value this large could occur randomly. The *P* value of less than 0.05 indicates that the model terms were also significant. Furthermore, the value of pure error was low, which indicates good reproducibility of the obtained data, with a low *P* value from the ANOVA and a satisfactory coefficient of determination (Table 2). The total determination coefficient (*R* ^2^) was 0.9123, which indicates that 91.23% of the variability in the response variables was explained and only 8.77% of the total variation was not explained using the model. The *R* ^2^ indicates the observed variability in the data that was accounted for by using the model. The adjusted *R* ^2^ (adj. *R* ^2^) modifies the *R* ^2^ by taking into account the number of covariates or predictors in the model [24]. The adj. *R* ^2^ was 0.8334, which suggests that there are excellent correlations between the independent variables. Several studies have supported the acceptance of any model with an *R* ^2^ value >0.75 [25, 26]. The significance of each coefficient, which was determined using the *F*-test, and *P* values are shown in Table 2. The corresponding variables would have been more significant if the absolute *F*-value was greater and the *P* value was smaller [27]. Table 2 shows that the linear terms *X*_1_ and *X*_3_, all quadratic terms, and interaction terms *X*_1_ *X*_2_ and *X*_1_ *X*_3_ had significant effects (*P* < 0.001 or *P* < 0.05), whereas the linear term *X*_2_ and interaction term *X*_2_ *X*_3_ did not have significant effects on LPS-induced PGE_2_ production (*P* > 0.05).

### 3.4. Canonical Analysis of the Stationary Point

Canonical analysis is a mathematical approach used to locate the stationary point of the response surface in the experimental region and to determine whether it represents a maximum, minimum, or saddle point [28]. The canonical form of the fitted LPS-induced PGE_2_ production is shown by using

(3) Y1=53.826529+29.048986ω12+15.900304ω22+11.573887ω32,

where *ω*_1_, *ω*_2_, and *ω*_3_ denote the transformed independent variables or the canonical variables. All the eigenvalues from (3) are positive, which indicates a unique minimum LPS-induced PGE_2_ production at the stationary point.

**Figure 1:**
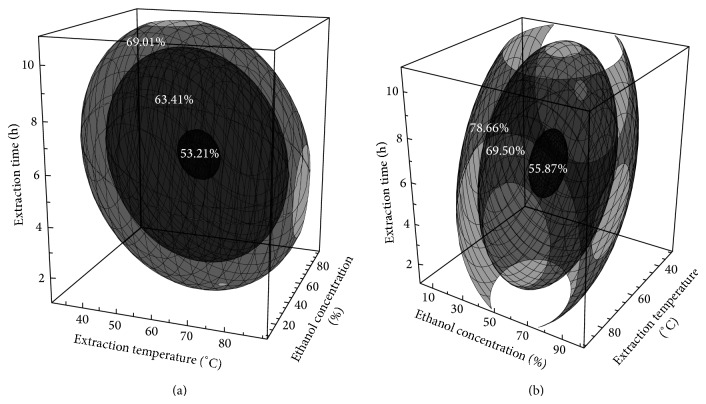
The 4-dimensional response surface plots showing the combined effect of extraction temperature, ethanol concentration, and extraction time on LPS-induced PGE_2_ (a) and NO production (b).

In the graphical approach, the predictive model for the LPS-induced PGE_2_ production was modified and used to create the four-dimensional response surface within the experimental region using a Mathematica program. As shown in Figure 1(a), the LPS-induced PGE_2_ production decreased as the extraction temperature and extraction time minimally increased (extraction temperature, 62°C; extraction time, 6 h; and ethanol concentration, 53%); therefore, the predicted stationary point was at the minimum (53.8%). The LPS-induced PGE_2_ production increased as the extraction temperature, extraction time, and ethanol concentration were increased above the minimum values. The ethanol extract of* A. capillaris* has been shown to have high levels of antioxidant activity [29] and to contain several other bioactive flavonoids, including scoparone, capillarisin, cirsimaritin, genkwanin, and rhamnocitrin [30]. It has been reported that the ethanol extract of* A. capillaris* exerted an anti-inflammatory effect on the mRNA expression level of cyclooxygenase-2 (COX-2) in LPS-stimulated RAW 264.7 cells [31]. COX-2 is an enzyme that generates prostaglandins, which are induced by proinflammatory cytokines and other activators, such as LPS, resulting in the release of a large amount of PGE_2_ at inflammation sites [32]. PGE_2_ is a major inflammatory lipid mediator involved in the pathogenesis of chronic inflammatory diseases such as rheumatoid arthritis, and it is synthesized by macrophages and other cell types in the presence of LPS [33, 34]. These results suggest that the extraction conditions might influence the decrease in PGE_2_ production in LPS-induced RAW 264.7 cells and provide a crude extract with high anti-inflammatory activity. The beneficial effects of nonsteroidal anti-inflammatory drugs (NSAID) in the treatment of inflammatory diseases have been well documented in the last decades. Evidence exists that these effects are mediated by an effective limitation of the production of PGE_2_ at the site of inflammation [35].

### 3.5. Optimization of LPS-Induced NO Production

Recently, suppression of the level of NO generation has been emphasized as a new pharmacological strategy for the treatment of inflammation-related diseases [19]. NO production may reflect the degree of inflammation and provides a measure to assess the effect of chemopreventive agents on the inflammatory process.

### 3.6. Model Fitting and Statistical Analysis

On the basis of the experimental results of CCD (Table 1) and regression analysis, an equation for the response surface was developed to estimate the relationship between the LPS-induced NO production and the independent variables (*X*_1_, *X*_2_, and *X*_3_). The model could be expressed as follows:

(4) NO=175.977805−2.059834X1−0.528652X2−12.992655X3+0.016046X12+0.010313X22+0.686408X32−0.007997X1X2+0.074690X1X3−0.009278X2X3.

The results of the ANOVA for the adequacy and fitness of the models are summarized in Table 2. The data indicated that the proposed regression model for the LPS-induced NO production was adequate with a satisfactory *R* ^2^ value (determined coefficient). The *R* ^2^ value for the LPS-induced NO production was 0.9390, which showed a close agreement between the experimental results and the theoretical values predicted by the polynomial model. These results, along with the high model *F*-value of 17.10, imply that the predicted model for the LPS-induced NO production was significant (*P* < 0.0001) and adequate. The *P* values are used as a tool to check the significance of each coefficient. The smaller the magnitude of the *P* value is the more significant the corresponding coefficient is, and this strongly affects the response variable [36]. From Table 2, the quadratic term of the ethanol concentration (*X*_2_^2^) had the largest effect on LPS-induced NO production, as indicated by its lowest *P* value (<0.0001) and highest absolute *F*-value (9.14). Next, the linear terms of the extraction temperature (*X*_1_) and extraction time (*X*_3_) show a substantial significant effect at a 99.9% confidence level (*P* < 0.001), and the interaction term *X*_1_ *X*_2_ were significant (*P* < 0.01).

### 3.7. Canonical Analysis of the Stationary Point

The canonical analysis revealed that the stationary point was a minimum and the canonical form of the fitted response model could be depicted by the equation

(5) Y2=56.069620+29.351142ω12+19.055285ω22+8.978654ω32.

As the eigenvalues were all positive, the stationary point was a minimum. On the basis of the predicted model, a four-dimensional response surface for LPS-induced NO production is shown in Figure 1(b). LPS-induced NO production had a minimum predicted value at 56%, obtained under the following conditions: 62°C extraction temperature, 52% ethanol concentration, and 6 h extraction time. On the basis of the results shown in Figure 1(b), at low extraction temperatures and a short extraction time, the LPS-induced NO production first decreased and then increased with increasing ethanol concentration, suggesting that an intermediate ethanol concentration is favorable. NO is the key regulator of immune responses and is involved in various physiological and pathological processes. Therefore, NO is a potential target for new therapeutic strategies, and the suppression of NO production has been emphasized for the treatment of inflammation-related diseases [19]. During an inflammatory response, a proinflammatory gene such as inducible nitric oxide synthase (iNOS) is catalyzed through signal transduction pathways leading to NO production [37]. Lim et al. [31] reported that the ethanol extract of* A. capillaris* suppressed NO production via the downregulation of iNOS transcription. The mRNA and protein levels of iNOS were suppressed markedly by the ethanol extract of* A. capillaris* treatment in LPS-stimulated RAW 264.7 cells. Therefore, the ethanol extract of* A. capillaris* could be a good raw material for the development of drugs for the treatment of chronic inflammatory diseases [31]. These results demonstrate that the extraction conditions may contribute to the anti-inflammatory activity of* A. capillaris* in LPS-induced RAW 264.7 cells and may effectively yield a crude extract with high anti-inflammatory activity.

### 3.8. Prediction and Verification of Optimum Extraction Conditions

**Figure 2:**
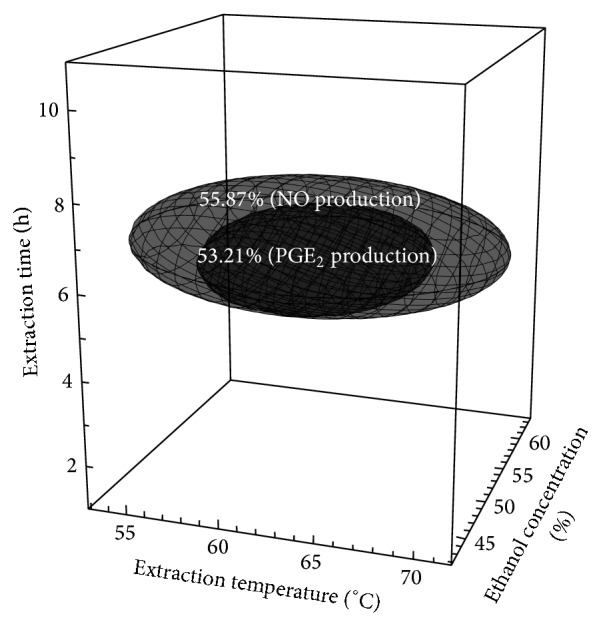
Superimposed response surface plots of the LPS-induced PGE_2_ and NO production showing optimal conditions for obtaining the extracts (*Artemisia capillaris* Thunb.).

On the basis of the above findings, an optimization study was performed to evaluate the optimal extraction conditions for individual responses as well as the combination of all responses. First, the optimum ranges for extraction parameters of* A. capillaris* Thunb. were predicted by superimposing the 4-dimensional response surface plots of the LPS-induced PGE_2_ and NO production (Figure 2). The ranges of extraction conditions used for determination of the optimal conditions were extraction temperatures of 57–67°C, ethanol concentrations of 46–60%, and extraction times of 5.2–7.0 h.

### 3.9. Cell Viability

Cell culture can be used to screen for toxicity both by estimation of the basal functions of the cell (i.e., processes common to all types of cells) or by tests on specialized cell functions [32]. Cell viability assays are used to identify the lack of certain toxic properties in the early stages of the development of potentially useful new substances such as therapeutic drugs, agricultural chemicals, and food additives. Therefore, a cytotoxicity test is a scientific analysis of the effects of toxic chemical substances on cultured mammalian cells. Cytotoxicity should be considered as a response variable in addition to LPS-induced PGE_2_ and NO production. The model adequacy of cytotoxicity was also predicted through regression analysis and ANOVA. The results of the ANOVA *R* ^2^ and adj. *R* ^2^ were 0.8941 and 0.7988, respectively (Table 2). The model could be expressed as follows:

(6) Cytotoxicity=72.885069+0.320093X1−0.037478X2+3.116604X3−0.004113X12+0.002561X22−0.306064X32+0.002755X1X2+0.002083X1X3+0.017361X2X3.

The results of the canonical analysis revealed that the stationary point was the maximum and cytotoxicity showed the maximum predicted value at 91.1% at 56°C extraction temperature, 45% ethanol concentration, and 6.5 h extraction time.

**Figure 3:**
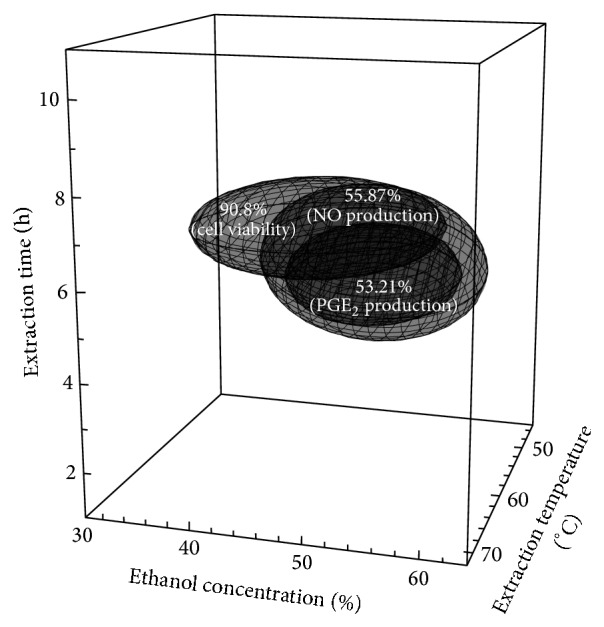
Superimposed response surface plots of cytotoxicity and LPS-induced PGE_2_ and NO production showing optimal conditions for obtaining the extracts (*Artemisia capillaris* Thunb.).

Finally, the optimum ranges for the extraction parameters were predicted by superimposing the 4-dimensional response surface plots of LPS-induced PGE_2_ and NO production and by cytotoxicity of* A. capillaris* Thunb. extracts (Figure 3). The ranges of extraction conditions used for the determination of the optimal conditions were an extraction temperature of 57–65°C, ethanol concentrations of 45–57%, and an extraction time of 5.5–6.8 h. In order to validate the predicted optimal extraction conditions for both components, an optional midpoint for each condition was selected within the ranges, that is, extraction temperature of 61°C, ethanol concentration of 51%, and extraction time of 6.2 h (Table 3). The experimental results for the LPS-induced PGE_2_ and NO production were 52.65 ± 1.01% and 57.55 ± 1.23%, respectively, and the results were in close agreement with the predicted values (LPS-induced PGE_2_ production: 53.87%; LPS-induced NO production: 56.16%) based on a response regression within 95% confidence intervals of the experimental values. As a result, the model from a central composite design was considered to be accurate and reliable for predicting the reduction in the LPS-induced PGE_2_ and NO production of extracts at the cellular level.

### 3.10. HPLC Analysis

**Figure 4:**
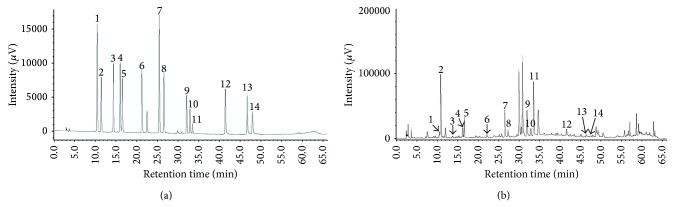
Typical HPLC chromatogram of phenolic and flavonoid (a) standards and (b) compounds in the* Artemisia capillaris* Thunb. extract at a point selected within the optimal ranges (extraction temperature, 62°C; ethanol concentration, 53%; extraction time, 6.1 h). Peaks: 1, chlorogenic acid; 2, catechin; 3, caffeic acid; 4, epicatechin; 5, epigallocatechin gallate; 6, coumaric acid; 7, rutin; 8, catechin gallate; 9, naringin; 10, apigenin-7-glucoside; 11, hesperidin; 12, quercetin; 13, apigenin; 14, kaempferol.

A typical HPLC chromatogram of phenolic and flavonoid compounds in the* A. capillaris* Thunb. extract at a point selected within the optimal ranges (extraction temperature, 62°C; ethanol concentration, 53%; extraction time, 6.1 h) is presented in Figure 4. The amount of selected phenolic and flavonoid compounds detected in the analyzed samples is shown in Table 4. Results are expressed in milligrams per g of dry sample.

## 4. Conclusion

This study indicates that the effects of extraction temperature, ethanol concentration, and extraction time on anti-inflammatory activities were significant, and the predicted second-order polynomial models of the LPS-induced PGE_2_ and NO production and cytotoxicity in RAW 264.7 cells were also significant and suitable. The optimal conditions determined by superimposing the 4-dimensional response surface plots of all the responses (*Y*_1_, *Y*_2_, and *Y*_3_) were as follows: an extraction temperature of 57–65°C, ethanol concentration of 45–57%, and extraction time of 5.5–6.8 h. The present study is, to the best of our knowledge, the first to establish the optimal extraction conditions for improving the anti-inflammatory activity of* A. capillaris* Thunb. by utilizing the response variables from* in vitro* analysis and RSM.

## Figures and Tables

**Table 1:** The central composite experimental design and experimental data for the optimization of conditions for obtaining  *Artemisia capillaris* Thunb. extracts.

Run	Independent variables^1^			Response variables^2^		
	*X*_1_ (°C)	*X*_2_ (%)	*X*_3_ (h)	*Y*_1_ (%)	*Y*_2_ (%)	*Y*_3_ (%)
1	42 (−1)	20 (−1)	3 (−1)	73.38 ± 0.21	79.18 ± 1.19	87.34 ± 2.04
2	78 (1)	20 (−1)	3 (−1)	78.81 ± 0.15	83.32 ± 0.24	84.98 ± 4.38
3	42 (−1)	80 (1)	3 (−1)	86.69 ± 0.13	87.21 ± 0.33	80.36 ± 0.52
4	78 (1)	80 (1)	3 (−1)	67.11 ± 0.23	69.34 ± 0.32	81.46 ± 0.89
5	42 (−1)	20 (−1)	9 (1)	74.38 ± 0.58	69.55 ± 1.06	85.98 ± 1.19
6	78 (1)	20 (−1)	9 (1)	85.69 ± 0.79	85.08 ± 0.88	82.56 ± 1.34
7	42 (−1)	80 (1)	9 (1)	68.32 ± 0.25	69.50 ± 0.24	82.88 ± 1.34
8	78 (1)	80 (1)	9 (1)	73.38 ± 0.14	72.50 ± 0.16	86.92 ± 2.15
9	30 (−1.682)	50 (0)	6 (0)	75.15 ± 0.42	78.66 ± 0.24	89.89 ± 0.41
10	90 (1.682)	50 (0)	6 (0)	63.41 ± 0.11	63.18 ± 0.31	88.13 ± 2.75
11	60 (0)	0 (−1.682)	6 (0)	71.65 ± 0.52	80.68 ± 0.24	85.24 ± 0.37
12	60 (0)	100 (1.682)	6 (0)	75.15 ± 0.81	83.84 ± 0.16	85.50 ± 1.41
13	60 (0)	50 (0)	1 (−1.682)	69.01 ± 0.19	75.92 ± 0.27	80.28 ± 1.30
14	60 (0)	50 (0)	11 (1.682)	66.71 ± 0.14	71.36 ± 0.32	88.00 ± 0.26
15	60 (0)	50 (0)	6 (0)	53.21 ± 0.21	56.50 ± 0.98	90.47 ± 1.26
16	60 (0)	50 (0)	6 (0)	54.14 ± 0.47	56.03 ± 1.79	91.20 ± 1.15
17	60 (0)	50 (0)	6 (0)	54.36 ± 0.23	56.03 ± 0.86	90.75 ± 1.89
18	60 (0)	50 (0)	6 (0)	54.75 ± 0.15	57.12 ± 0.28	90.13 ± 0.74
19	60 (0)	50 (0)	6 (0)	53.97 ± 0.50	56.65 ± 0.91	91.75 ± 1.71
20	60 (0)	50 (0)	6 (0)	54.36 ± 0.14	55.87 ± 1.07	90.13 ± 2.34

^1^Independent variables: *X*_1_, extraction temperature; *X*_2_, ethanol concentration; *X*_3_, extraction time.

^2^Response variables: *Y*_1_, LPS-induced PGE_2_ production; *Y*_2_, LPS-induced NO production; *Y*_3_, cytotoxicity.

**Table 2:** Regression coefficients of the predicted second-order polynomial models and the results of an analysis of variance for LPS-induced PGE_2_ and NO production and cytotoxicity in RAW 264.7 cells.

Source	LPS-induced PGE_2_ production			LPS-induced NO production			Cytotoxicity		
	Coefficients	*F*-value	*P* value	Coefficients	*F*-value	*P* value	Coefficients	*F*-value	*P* value
*β*_0_	173.194583	8.42	<0.0001	175.977805	9.90	<0.0001	72.885069	9.10	<0.0001
Linear									
*β*_1_	−2.523157	−5.11	0.0005	−2.059834	−4.82	0.0007	0.320093	1.66	0.1274
*β*_2_	−0.289006	−1.19	0.2625	−0.528652	−2.51	0.0308	−0.037478	−0.40	0.7010
*β*_3_	−11.023825	−4.38	0.0014	−12.992655	−5.97	0.0001	3.116604	3.18	0.0099
Quadratic									
*β*_11_	0.019932	5.50	0.0003	0.016046	5.12	0.0005	−0.004113	−2.91	0.0155
*β*_22_	0.008825	6.76	<0.0001	0.010313	9.14	<0.0001	−0.002561	−5.04	0.0005
*β*_33_	0.660889	5.06	0.0005	0.686408	6.08	0.0001	−0.306064	−6.02	0.0001
Interaction									
*β*_1_ *β*_2_	−0.007467	−2.59	0.0268	−0.007997	−3.21	0.0093	0.002755	2.45	0.0340
*β*_1_ *β*_3_	0.072940	2.53	0.0298	0.074690	3.00	0.0133	0.002083	0.19	0.8564
*β*_2_ *β*_3_	−0.029131	−1.69	0.1228	−0.009278	−0.62	0.548	0.017361	2.58	0.0275
SSPE^1^	1.366898			1.129981			1.635000		
Total model		11.56	0.0003		17.10	<0.0001		16.97	0.0037
*R* ^2^	0.9123			0.9390			0.8941		
Adjusted *R* ^2^	0.8334			0.8841			0.7988		

^1^Pure error of the sum of squares.

**Table 3:** Optimal extraction conditions determined by superimposing the response surfaces for extracts from *Artemisia capillaris* Thunb.

Independent variables	Optional condition (predicted ranges)	Predicted value		Experimental value	
		*Y*_1_^4^	*Y*_2_^5^	*Y*_1_	*Y*_2_
*X*_1_^1^	61 (57–65)	53.87	56.16	52.65 ± 1.01	57.55 ± 1.23
*X*_2_^2^	51 (45–57)				
*X*_3_^3^	6.2 (5.5–6.8)				

^1^ *X*_1_: extraction temperature (°C).

^2^ *X*_2_: ethanol concentration (%).

^3^ *X*_3_: extraction time (h).

^4^ *Y*_1_: LPS-induced PGE_2_ production (%).

^5^ *Y*_2_: LPS-induced NO production (%).

**Table 4:** Contents of selected phenolic and flavonoid compounds in the *Artemisia capillaris* Thunb. extract at a point selected within the optimal ranges (extraction temperature, 62°C; ethanol concentration, 53%; extraction time, 6.1 h).

	Compounds	Contents (mg g^−1^)^1^
Phenolic compounds	Chlorogenic acid	1.43 ± 0.10
	Caffeic acid	0.16 ± 0.01
	Coumaric acid	0.21 ± 0.03

Flavonoids	Rutin	8.33 ± 0.95
	Naringin	6.69 ± 0.70
	Apigenin-7-glucoside	2.79 ± 0.63
	Hesperidin	57.44 ± 7.14
	Quercetin	1.98 ± 0.12
	Apigenin	3.13 ± 0.53
	Kaempferol	0.51 ± 0.04

Catechins	Catechin	51.76 ± 6.83
	Epicatechin	2.60 ± 0.52
	Epigallocatechin gallate	12.29 ± 1.60
	Catechin gallate	2.61 ± 0.48

^1^Values are mean ± SD of triplicate determinations.
